# Medical Association of Ala.

**Published:** 1886-05-20

**Authors:** 


### MEDICAL ASSOCIATION OF ALABAMA.

TO EDITORS OF THE RECORD:

The Medical Association of the State of Alabama met in the city of Anniston April 13, at 12 o'clock m.—Dr. F. M. Peterson in the chair.

The meeting was largely attended, and in many respects the most interesting session since the organization of the Association.

Drs. Taliaferro and Love, of Atlanta, and Dr. Battey, of Rome, Ga, were at the meeting.

Dr. Sealy, of Montgomery, was elected president, and Dr. Robertson, of Oxford, vice-president, for the ensuing year.

The next annual meeting will be held at Tuscaloosa, in April, 1887.

